# Incidence of lower limb lymphedema after vulvar cancer: A systematic review and meta-analysis

**DOI:** 10.1097/MD.0000000000008722

**Published:** 2017-11-17

**Authors:** Jiuzuo Huang, Nanze Yu, Xiaojun Wang, Xiao Long

**Affiliations:** Division of Plastic and Reconstructive Surgery, Peking Union Medical College Hospital, Beijing, China.

**Keywords:** lymphedema, meta-analysis, vulvar cancer

## Abstract

**Background:**

Lower limb lymphedema (LLL) is an important concern for patients with vulvar cancer. Studies of the incidence of vulvar cancer-related lymphedema and its risk factors have substantially increased in the new millennium.

**Objectives:**

This article is a meta-analysis that aimed to systematically evaluate the incidence of LLL and its risk factors related to vulvar cancer.

**Data sources:**

Data were collected from eligible studies from PubMed, ScienceDirect, and Web of Science.

**Synthesis methods:**

Random effects models were used to calculate a pooled overall estimate of LLL incidence, and subgroup analyses were performed to assess the effects of different study designs, countries of study origin, diagnostic methods, and extent of lymph node surgery. Risk factors for lymphedema were also evaluated.

**Results:**

Twenty-seven studies met the inclusion criteria for the assessment of lymphedema incidence with a pooled estimate of 28.8% [95% confidence interval (CI) 22.1–35.5]. The estimate was 16.7% (95% CI 9.7–23.7) when data were restricted to prospective cohort studies (7 studies). The incidence of LLL was increased by approximately 5-fold in women who underwent inguinofemoral lymph node dissection compared to those who underwent sentinel lymph node biopsy. The reported risk factors included wound infection, inguinofemoral lymphadenectomy, older age, body mass index (BMI), and radiation therapy.

**Conclusions:**

Approximately 3 in 10 women who survive vulvar cancer will develop lower limb lymphedema. More studies are needed to improve the understanding of its risk factors and to develop prevention and management strategies to alleviate this distressing disorder.

## 1 Introduction

Lower limb lymphedema (LLL) is one of the most disabling side effects of surgical treatment for gynecological cancer and is characterized by regional swelling, typically in one or both lower limbs, caused by excess accumulation of protein-rich fluid in body tissues.^[1]^ Symptoms of LLL include leg heaviness, itching, pain, skin changes, infection, decreased mobility, and negative self-image.^[2]^ Although LLL can occur after treatment of ovarian, uterine, and cervical cancers, the incidence of LLL is highest after treatment of vulvar cancer.^[3]^

Vulvar cancer is a disease with increasing incidence,^[4]^ particularly among younger women^[5]^; therefore, LLL is a disease that is also increasing in prevalence given the longer life expectancy of these women. Understanding the incidence of LLL is clearly of public health importance.

Individual studies report wide variations in the incidence of LLL (0%–73%),^[6,7]^ which is an indication of the differences in study design, diagnostic methods and criteria used and the timing of lymphedema measurement with respect to vulvar cancer diagnosis and treatment. Some estimates suggest that approximately 30% of women will develop LLL after vulvar cancer. This estimation is the average incidence of studies that have been included in several reviews of lymphedema after vulvar cancer.^[8,9]^ However, the average incidence of a group of studies does not consider factors that are known to affect detection rates, such as the study design or timing and method of lymphedema assessment. Therefore, the incidence of lymphedema after vulvar cancer is unclear.

The body of evidence related to the incidence of LLL after vulvar cancer has grown substantially and has improved in quality during the past decade, now including findings from several prospective cohort studies. We therefore performed this systematic review and meta-analysis to provide the most up-to-date estimate of the incidence and risk factors of LLL after vulvar cancer.

## 2 Methods

We followed the recommendations for interventional reviews provided in the Cochrane Handbook (version 5.1.0); our work was Assessment of Multiple Systematic Reviews (AMSTAR)-compliant, and our report was guided by the principles outlined in the Preferred Reporting Items for Systematic Reviews and Meta-Analysis (PRISMA) statement.^[10]^ As it was a systematic review, it did not require ethical approval or patient consent.

### 2.1 Search strategy and selection criteria

We performed a systematic review to identify all studies addressing vulvar cancer-related LLL. We performed a comprehensive search of databases, including Academic Search Elite, Cumulative Index to Nursing and Allied Health, Cochrane Central Register of Controlled Trials (clinical trials), PubMed, ScienceDirect, and Web of Science, to identify studies published between January 1, 2000 and December 31, 2016 that included women who underwent surgery for vulvar cancer. The search terms included keywords for vulvar cancer (“vulvar” or “vulva” and “cancer” or “onco∗” or “neoplasm∗”) and lymphedema (“lymphedema” or “lymphedema”).

The eligibility criteria for inclusion of studies in this review and meta-analysis comprised 7 categories. For the type of study: published research articles were included; review articles, meta-analyses, editorial or comment articles, and case reports were excluded. Patient characteristics: studies of female patients with vulvar cancer were included; studies of patients with primary lymphedema or metastatic disease were excluded. Diagnosis of lymphedema: self-reported swelling was the only symptom considered an indication of self-reported lymphedema; studies that reported the incidence of lymphedema only on the basis of multiple symptoms (eg, “do you have pain, tingling, or weakness of the leg?”) were excluded because these symptoms are common regardless of lymphedema status, and the inclusion of such symptoms might therefore lead to an overestimation of lymphedema incidence. All objective methods of diagnosing lymphedema were included. Outcome included the incidence of, prevalence of, or risk factors for secondary lymphedema were included. In the absence of pretreatment lymphedema status, prevalence was considered a reasonable estimate of incidence because the proportion of women with lymphedema before surgery for vulvar cancer has been reported to be very low. Time period included outcome data measured within 4 weeks after surgery were excluded because lower extremity-related changes during this timeframe were considered potentially indicative of an acute treatment-related response. For language and origin, we included studies available from all locations with reports written in English; when translations were unavailable, non-English-language articles were excludedStudies had a sample size of at least 10 patients.

### 2.2 Data extraction

One investigator (JH) selected articles that potentially met our inclusion criteria based on their titles and abstracts. Full articles were then retrieved for a more detailed assessment. We developed a data abstraction sheet to collect necessary information to establish the level of evidence (defined by the Oxford Centre for Evidence-Based Medicine), and available outcome and risk factor details. For each included study, 1 investigator (JH) extracted data regarding the study location (country), study design, sample size, method of lymphedema assessment, definition of lymphedema, incidence or prevalence of lymphedema, and any risk factor information. Study designs included randomized controlled trials, cross-sectional, prospective cohorts, retrospective cohorts, and case-control studies. Case-control studies were not included for lymphedema incidence estimation. Lymphedema measurement refers to the technique used to define the presence of lymphedema and included bioimpedance spectroscopy, leg circumferences, water displacement or perometry (optoelectronic volumeter), lymphoscintigraphy, clinician diagnosis, and patient-reported diagnosis by a clinician or self-reported swelling.

We categorized all studies that analyzed the incidence of leg lymphedema into levels of evidence based on the study design using the levels of evidence (Prognosis column) defined by the Oxford Centre for Evidence-Based Medicine (CEBM). Two investigators (JH and NY) independently categorized each study, and disagreements were resolved through discussion with a third assessor (XL) to attain a consensus.

We assessed the presence of publication bias using funnel plots. The funnel plot was symmetrical regarding the summary effect, with larger studies at the top and smaller studies at the bottom, indicating no clear evidence of publication bias.

### 2.3 Statistical analyses

The main outcome of interest for this analysis was the overall cumulative incidence (%) of LLL after the diagnosis or treatment of vulvar cancer, which was obtained from the published reports of incidence or prevalence. Exact binomial 95% CIs were subsequently calculated. When incidence was presented separately by treatment (eg, sentinel lymph node biopsy vs lymph node dissection), we calculated an overall incidence for the study using data from the reports. *I*^*2*^ tests were performed to evaluate heterogeneity. Fixed effects models were used in the analysis if no substantial heterogeneity was present (*P* > .10) among different studies; otherwise, the random effects model was applied (*P*≤ .10). All analyses were conducted using the Comprehensive Meta-analysis (version 2).

## 3 Results

**Figure 1:**
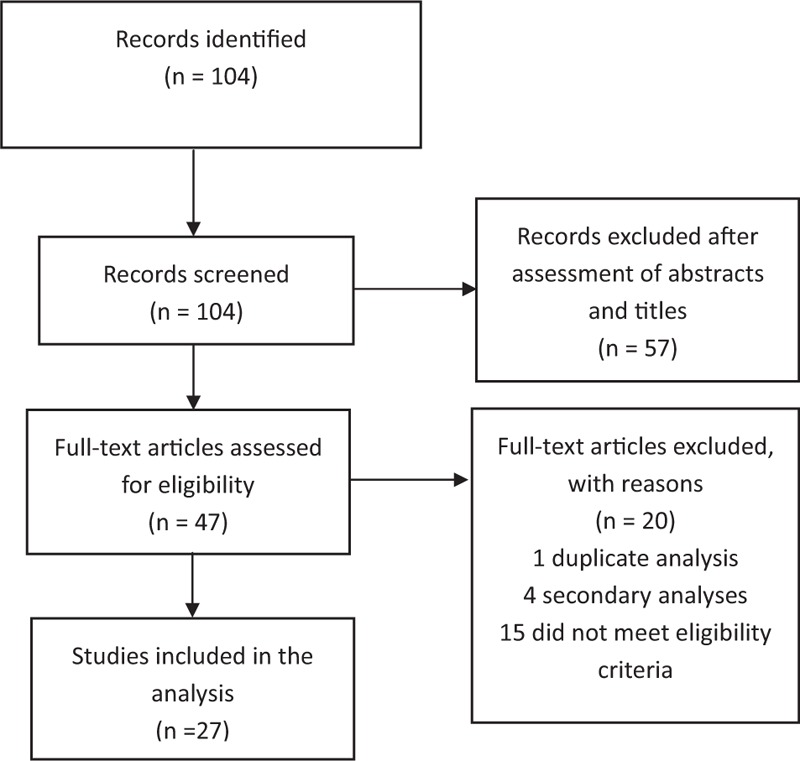
Flow diagram indicating the literature search and selection process, which were conducted in accordance with the Preferred Reporting Items for Systematic Reviews and Meta-Analysis statement.

In total, 104 potentially relevant citations were identified, of which 27 articles were included in our analysis (Fig. 1).^[6,11–36]^

Most studies were either retrospective cohort or prospective cohort studies, but we also identified cross-sectional studies and case randomized control trials. Approximately half of the studies were conducted in Europe, and the most common method of lymphedema measurement was clinical diagnosis.

We calculated a pooled estimate of LLL incidence of 28.8% (95% CI 22.1–35.5) using data obtained from 27 studies of 2535 women with vulvar cancer (Table 1). The incidence ranged between 16.7% and 49.2%, with cross-sectional studies showing the highest estimate and prospective cohort studies showing the lowest estimate.

**Table 1:**
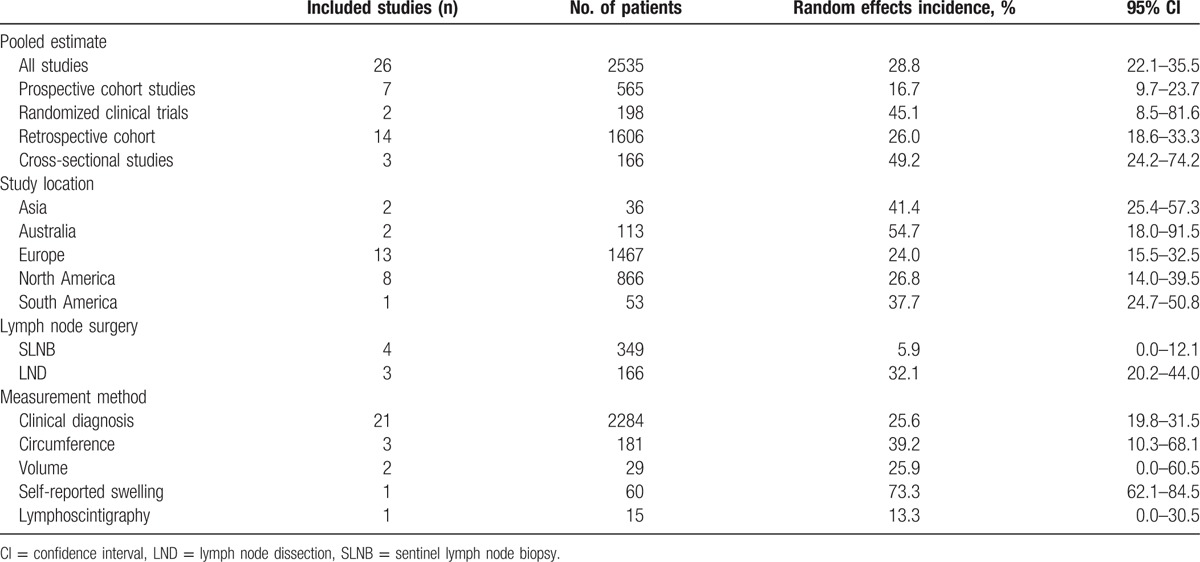
Incidence of vulvar cancer-related lymphedema.

The lowest incidence was observed in Europe, and the highest incidence was noted in Australia. The incidence of LLL in women who underwent inguinofemoral lymph node dissection was increased approximately 5-fold compared with those who underwent sentinel lymph node biopsy.

Clinical diagnosis (21 studies) was the most common type of method used to diagnose lymphedema. Circumference (3 studies) and volume (2 studies) were also performed for lymphedema evaluation. The highest estimates were reported by 1 study that used self-reported swelling to classify lymphedema, whereas 1 study that classified lymphedema according to lymphoscintigraphy reported the lowest incidence.

Seven studies investigated risk factors (Table 2).^[11–24,27,29–36]^ Wound infection, inguinofemoral lymphadenectomy, and older age were validated as risk factors in 2 studies, whereas BMI and radiation therapy were noted as risk factors in 1 study.

**Table 2:**
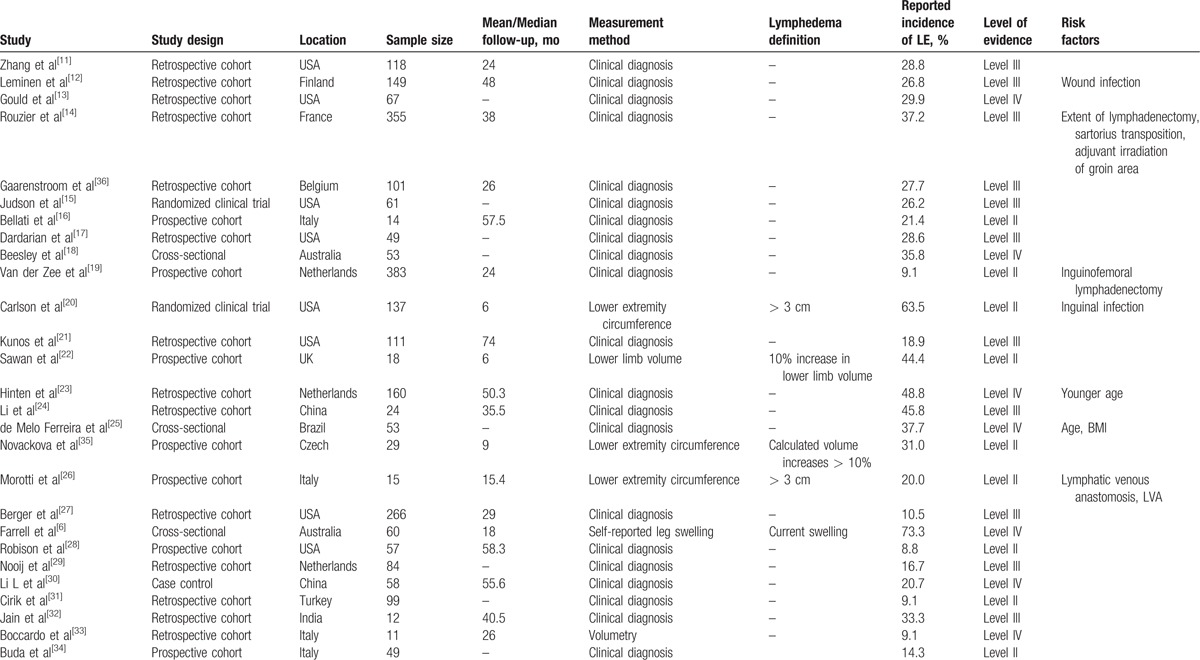
Studies reporting lymphedema incidence or prevalence after vulvar cancer treatment and associated risk factors.^[11–24,27,29–36]^.

## 4 Discussion

Our findings suggest that 16.7% of women with vulvar cancer will develop LLL. These findings are based on data from prospective cohort studies, which are well-suited for assessing incidence.

Although similar to reports from previous reviews,^[8]^ the subgroup analyses reported here provide additional insight. First, approximately 1 in 4 women with vulvar cancer living in Europe and North America, 1 in 3 women living in Asia and South America, and approximately 1 in 2 women living in Australia develop LLL. Second, LLL is approximately 5-fold more likely to occur when inguinofemoral lymph node dissection is used than when the more conservative sentinel lymph node biopsy procedure is used. Finally, evidence lends support to several risk factors for LLL, including wound infection, inguinofemoral lymphadenectomy, older age, BMI, and radiation therapy. These factors are potential targets for future prevention strategies or for more effective management of the disorder.

The accuracy of our estimate of LLL incidence should be considered. Missing data might affect incidence estimates, but the direction of the bias is unknown. The effect of the potential publication bias and attrition bias on the results must also be considered. For example, reports with a very high incidence of lymphedema might not be published because the “clinical data are not good enough.” Different study designs might have various attrition biases. Prospective cohort studies are optimal for evaluating the incidence of LLL. In contrast, cross-sectional studies provide information about prevalence, which is the proportion of individuals who have a disease or condition at one point in time. Retrospective studies rely on data recall or information available from records collected for other purposes. Theoretically, incidence estimates from prospective cohort studies should be higher than data derived from other types of studies. However, the incidence of lymphedema estimates is lowest from prospective cohort studies compared with other types of studies. One reason might be that most of the patients (350/565) received sentinel lymph node biopsy in the prospective studies, which results in a much lower lymphedema incidence than lymph node dissection. Furthermore, the prevalence of lymphedema is also related to the follow-up duration.

The type of method used to diagnose lymphedema also affects incidence data. Currently, no gold standard measurement or defining criteria exist for lymphedema assessment in the clinical setting, and the lack of a convenient, standardized, objective measurement method has resulted in reports of widely varying incidence. Although circumference measures may appear to be the most convenient method, several problems exist, including problems with control of intra- and intermeasurer reliability. The process is also time-consuming and requires considerable experience to obtain accurate measures. Although limb volume assessment using water displacement has historically been regarded as the most sensitive and accurate means to assess limb volume changes, clinicians rarely use this cumbersome approach outside the research setting. Noninvasive assessments of leg volume using lower limb circumferences at multiple levels might be a valuable adjuvant means of diagnosis for LLL.^[35]^

In addition to the aforementioned issues, other factors also influence the incidence of lymphedema, including the duration of follow-up, different procedures used for tumors and lymph nodes, stage of cancer, wound infection, BMI, chemotherapy, and radiation therapy. Different surgical techniques also influence the lymphedema incidence, including sartorius transposition,^[15]^ saphenous vein sparing,^[17]^ the triple incision technique,^[24]^ and lymphatic-venous anastomoses.^[33]^ A combination of the above issues could have affected the incidence estimate.

The strengths of this study include the large combined sample size for estimating the lymphedema incidence, subgroup analyses (for different study designs, locations of study, lymph node surgery, and measurement methods), and the use of meta-analytic techniques. Specific eligibility criteria were used to exclude studies with sample sizes less than 10 or outcome data measured within 4 weeks after surgery.

The weakness associated with this review can largely be attributed to the lack of consensus in the field of lymphedema. Given the lack of precise consensus definitions or grading systems for lymphedema, clinicians, and investigators have used a wide variety of independently developed, nonvalidated, subjective, and objective diagnostic and rating criteria. In addition, limited patient-level data related to patient characteristics (ie, BMI), radiation therapy, follow-up duration, and postoperative complications did not allow for adjusted risk analyses.

## 5 Conclusions

Approximately 3 in 10 women who survive vulvar cancer will develop LLL. Additional prospective cohort studies that incorporate validated instruments and objective measurements are needed to further define the magnitude of this condition.
